# Situational xylophagia from chronic anemia: case report

**DOI:** 10.3389/fpsyt.2025.1646188

**Published:** 2025-09-15

**Authors:** Jonathan Credo, Sumra Mubarik, Lorin M. Scher

**Affiliations:** ^1^ Department of Internal Medicine, Davis Health System, University of California, Davis, Sacramento, CA, United States; ^2^ Department of Psychiatry and Behavioral Sciences, Davis Health System, University of California, Davis, Sacramento, CA, United States; ^3^ Department of Chemistry and Biochemistry, Northern Arizona University, Flagstaff, AZ, United States; ^4^ Davis School of Medicine, University of California, Davis, Sacramento, CA, United States

**Keywords:** case report, xylophagia, Roux-en-Y bypass, microcytic anemia, iron deficiency

## Abstract

**Background:**

Xylophagia is a rare subtype of pica involving consumption of paper or wood products. It is unknown why an individual may choose non-food items for consumption; however, pica has higher co-occurrence in those with schizophrenia, intellectual disability, or mineral deficiency.

**Case presentation:**

We present a case of a patient with severe abdominal pain and a small bowel obstruction due to a paper bezoar (xylobezoar). Evaluation led to a diagnosis of xylophagia in the context of chronic symptomatic microcytic anemia and not due to an underlying primary psychiatric etiology. Anemia manifested in variable sensory experiences for the patient, including changes in the perception of smell, touch, and taste. The patient’s specific preference for books of a particular era could be influenced by changes in printing practices. Additionally, familial practices delayed recognition of xylophagia as an odd, learned behavior. Once recognized as untreated anemia and confirmed to have iron and zinc deficiency, oral supplementation was initiated throughout hospitalization with surgical surveillance following discharge.

**Conclusion:**

The presented patient developed a xylobezoar in the setting of severe symptomatic anemia precipitated by a combination of regular menses and a history of Roux-en-Y gastric bypass, leading to both monthly blood loss and chronic iron and zinc malabsorption, respectively. Although pica and xylophagia are frequently linked to primary psychiatric disorder or intellectual disability, and sometimes familial predisposition, the underlying trigger for these behaviors may, in some cases, stem from an unrecognized or untreated medical condition rather than a primary psychiatric disorder.

## Background

Xylophagia is a relatively rare subtype of pica that refers to an atypical compulsion to ingest paper and other products of wood. To diagnose an individual with any form of pica, they must meet the following criteria: (1) the behavior of ingesting non-nutritional, non-food items must occur for over one month, and (2) the behavior should be socially or culturally unacceptable (1). This unusual and maladaptive behavior has a higher co-morbid incidence in individuals with schizophrenia, obsessive compulsive disorder (OCD), learning disabilities including autism spectrum disorder (ACD) or intellectual disability (ID), or iron and zinc deficiencies (2). In patients with schizophrenia, OCD, ASD, and ID, it has been postulated that ritualistic and compulsive behaviors may function as self-regulatory mechanisms, serving to mitigate psychological distress and promote a sense of control. Populations with a relatively higher prevalence include young children, individuals from lower socioeconomic backgrounds, and pregnant women (2). While mouthing and oral fixation are typical behaviors seen in infants and young children, these behaviors are generally outgrown by early childhood. Various theories have been posited for why it first develops in children, ranging from developing sensory and psychological faculties to maladaptive coping strategies during periods of high stress or limited parental involvement (3). In patient populations with lower socio-economic status, consumption of non-food items has also represented a means to stretch food resources with low nutritional “fillers”, such as sawdust in bread flour (4, 5). Related, food adulteration, or the practice of replacing high value and nutritional food items with those of lower quality or even non-food items, has been practiced for economic gain, as seen in the spice trade, or when regulatory oversight is limited, including the distribution of adulterated food items to Native Americans or use of lead chromate in turmeric (5–7).

Although xylophagia is often seen as an odd or inappropriate behavior, it is essential to distinguish between psychiatric manifestations and behaviors that may result from untreated or underdiagnosed medical conditions. Pica has been associated with pregnancy, Celiac disease, gastric bypass operations, and nutritional deficiencies, particularly zinc and iron deficiency (8). In these instances, pica manifests as craving and a means to address physiological mineral deficiencies in non-food items that have increased iron or zinc content. The broad pathophysiological mechanism for these conditions is a result of either increased iron consumption (e.g., chronic anemia in women due to menses, pregnancy increased nutritional demand) or disruptions in nutritional absorption (e.g., Celiac disease, gastric bypass) (1, 8). Therefore, before attributing these behaviors to psychiatric causes, it is crucial to rule out potential underlying medical conditions. Additionally, it is necessary to also consider familial influence and reinforcement of such behaviors. Family members or caregivers may unknowingly contribute to the persistence of the behavior by providing attention or care when the behavior occurs. In this report, we will discuss the case of a patient diagnosed with xylophagia in the setting of iron-deficiency microcytic anemia (IDMA).

## Case presentation

**Figure 1:**
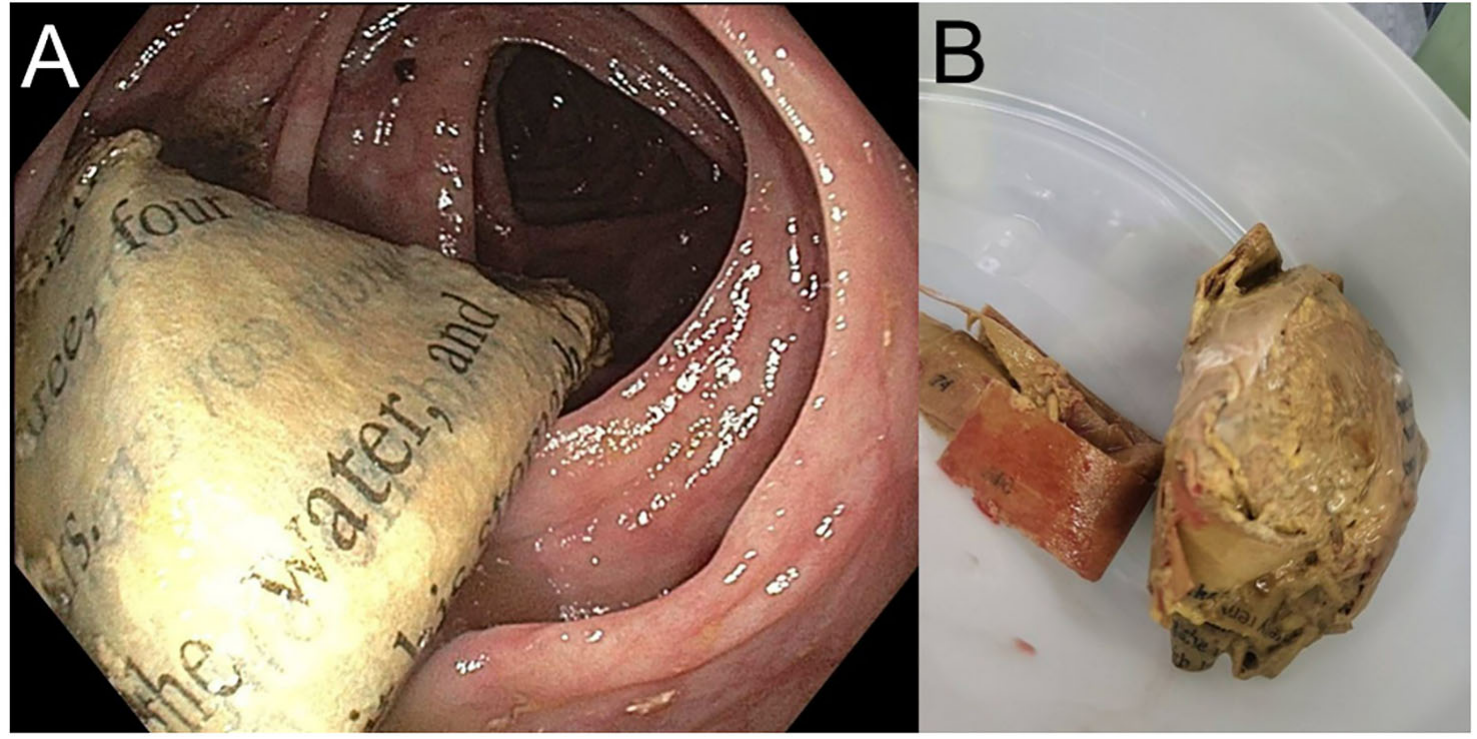
Xylobezoar, depicted internally **(A)** during exploratory colonoscopy and found within ascending colon and externally **(B)** after removal by surgical team. Both pictorial representations obtained by surgical services and without scale provided.

The inpatient psychiatry service was consulted by general surgery for a 40-year-old female identifying patient following emergent laparoscopic exploration and subsequent removal of a xylobezoar that was causing symptomatic small bowel obstruction (**Figure 1**). The consulting service was concerned that the patient’s presentation was due to an underlying untreated psychiatric condition. On initial evaluation, the patient’s mental status exam was notable for a pleasant affect with a linear and logical thought process, and intact cognition with a MoCA score of 27. Further, the patient demonstrated good insight into her condition when questioned about her behavior. The patient had a psychiatric history of generalized anxiety disorder (GAD) and major depressive disorder (MDD), with prior pharmacologic trials including paroxetine 10 mg, fluoxetine 20 mg, sertraline up to 50 mg, and citalopram 10 mg starting in 2011. At the time of presentation, she was maintained on doxepin (Sinequan) 100 mg at bedtime and vortioxetine (Trintellix) 10 mg each morning both since 2020 and methylphenidate extended release 45 mg daily for comorbid attention-deficit/hyperactivity disorder (ADHD) since 2023. Her relevant medical history included a Roux-en-Y gastric bypass in 2006, complicated by recurrent unspecified anemia beginning in 2008, for which she was on chronic oral iron supplementation.

The patient reported a 10-year history, beginning around 2014, of intermittent paper consumption occurring in the context of symptomatic anemia. She associated these episodes with symptoms of excessive fatigue, difficulty concentrating, and subjective increases in mood irritability. The patient was able to clearly differentiate these episodes from fatigue attributable to other causes, such as disrupted sleep due to caregiving responsibilities, regular physical activity, or intercurrent illness. She also did not attribute her paper consumption to her underlying diagnoses of GAD or MDD. Rather, she described her current psychiatric symptoms as primarily involving insomnia and ruminative thoughts characterized by guilt, unfulfilled aspirations, and feelings of isolation associated with her role as a homemaker. The patient reported that her psychiatric symptoms were well-managed for many years with her current pharmacologic regimen as noted above. When asked about potential triggers for her paper consumption, she identified that onset of menses – particularly during months of heavier menstrual flow – as a consistent precipitate of her cravings. Of note, she did not perceive the behavior as unusual, citing a familial context in which older female relatives, including maternal figures, reportedly consumed substances such as dirt to alleviate symptoms they associated with anemia. She denied any known history of diagnosed genetic, intellectual disability, or developmental conditions in any first- or second-degree relatives and only endorsed intermittent anxiety and depressive symptoms in her mother who lacked a formal diagnosis or treatment.

Of note, regarding her paper consumption, she described only wanting to consume books published before the year 2000 as these books had a specific aroma of “earthy-ness” and “richness” that was not present in modern books. The patient noted that while she generally found the scent of older books to be pleasant, this olfactory perception became markedly intensified during episodes of symptomatic anemia. The patient described a very fond instance of visiting a used bookstore and being surrounded by the pleasant aroma, so overwhelming that even the texture of the books was stimulating. The patient mused that following their current hospitalization, they would likely need to avoid these stores to avoid the compulsion to consume books. When screened for other obsessive-compulsive behaviors or oral fixation patterns, the patient denied any such tendencies. She reiterated that her paper consumption was not driven by symptoms of anxiety or depression but occurred exclusively during periods when she perceived herself to be anemic.

In the immediate 48-hour perioperative window, all psychiatric medications were held to limit possible clotting or surgical complications. After 48-hours, the primary surgical team and consultant psychiatry team agreed to restart the patient’s home doxepin 100mg at bedtime, vortioxetine 10mg every morning, and methylphenidate ER 45mg every morning. Initial labs drawn on admission demonstrated microcytic anemia with a hemoglobin of 9.6 g/dL and MCV of 63.7 fL, which was re-demonstrated following surgery where she received one unit packed red blood cells, presumably for intra-operative blood loss, with a hemoglobin of 10.0 g/dL and MCV of 65.1 fL. Total iron and zinc levels were recommended as laboratory add-ons by the psychiatry team 24-hours post-operative, both of which were low at 28 ug/dL iron and 16.7 ug/dL zinc. The patient consistently denied experiencing ongoing urges to consume paper products, acknowledging that this behavior was the likely precipitant of her current hospitalization. The patient was started on oral iron 18mg daily and zinc 50mg daily supplementation. IV iron replacement was withheld due to on-going global IV fluid shortages. At the time of discharge, the psychiatry consultation team determined that no modifications to the patient’s outpatient regimen were warranted. Following discharge from the hospital, the patient returned to their home health network which limited access to psychiatric and objective evaluation. Although, during a subsequent in-network surgical follow-up the patient received updated objective laboratory studies demonstrating on-going IDMA and reported seeing their established psychotherapist and psychiatrist with no further urges to consume paper products.

## Discussion and conclusion

**Figure 2:**
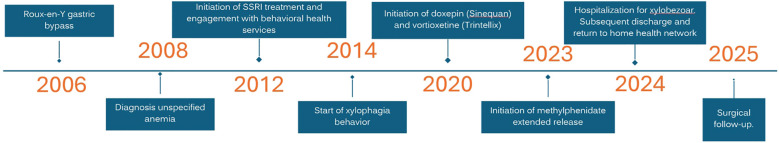
Timeline of events from Roux-en-Y gastric bypass to surgical follow-up.

The patient described in this report—a 40-year-old female with a history of Roux-en-Y gastric bypass and chronic IDMA—illustrates how underlying nutritional deficiencies can manifest as atypical eating behaviors, which might otherwise be misattributed to a primary psychiatric disorder. Psychiatric etiologies considered included decompensated GAD or MDD and OCD. However, the patient reported stability in their depressive and anxiety symptoms on their home regiment and did not endorse any obsessions or compulsions including oral fixations, although a Y-BOCS was not formally administered. The presumptive diagnosis of chronic IDMA is supported by their objective laboratory studies and the existence of prominent risk factors (e.g., Roux-en-Y gastric bypass and active menses) that limit absorption and increase consumption (**Table 1**). The timeline of the patient’s development of xylophagia causing IDMA is further supported by their timeline of events given iron deficiency can take up to two years to manifest on laboratory studies and longer before causing symptomatic manifestation (**Figure 2**) (9). Monthly menstruation periods are frequent causes for microcytic anemia in women and the association between heavier periods and symptomatic manifestation has been linked with the use of validated pictorial diagrams (10).

**Table 1:** Selected objective blood work laboratory studies from the patient during their hospitalization in October 2024 and at a repeat in-network hospital encounter in March 2025.

Date of study	28 October 2024	24 March 2025	Normal reference values
Hemoglobin (g/dL)	9.6	8.4	12.0 – 16.0
Hematocrit (%)	32.2	28.2	34.0 – 46.0
Mean Corpuscular Volume (MCV) (fL)	63.7	64.4	80.0 – 100.0
Serum iron (µg/dL)	28	14	37 – 158
Transferrin (mg/dL)	258	297	200 – 360
Total iron binding capacity (µg/dL)	359	413	280 – 400
Iron saturation (%)	7.8	3.4	15.0 – 50.0
Ferritin (ng/mL)		13	13.0 – 150.0
Zinc (µg/dL)	16.7		56.0 – 134.0

Selected objective studies include evaluation for anemia, studies included on hospital “iron studies panel” per hospital Department of Pathology and Laboratory Medicine, and zinc for nutritional evaluation.

A possible explanation and hypothesis for the patient’s preference for “older books” is due to changes in printing practices. Specifically, the metal content used in ink and paper manufacturing has steadily declined from the mid-20th century through the early 21st century. The cause for this shift in production practices included lower toxicity risks for producers, consumers, and the environment, stricter environmental regulations, increased energy and cost efficiency due to changes in printing technologies, and increased vibrancy of organic and synthetic based inks (11, 12). While we did not perform exhaustive cranial nerve testing, previous human studies have demonstrated individualistic thresholds for sensing metals by taste and smell including differences due to nasal occlusion and lipid content, including a potential gustatory and tactile link to iron content and purity (13, 14). In contrast to certain psychiatric conditions in which compulsive behaviors are driven by intrusive thoughts or anxiety, the paper consumption observed in this case was attributed to an underlying medical condition—specifically, IDMA. This nutritional distinction is clinically significant, as it suggests that addressing the underlying deficiency may serve as an effective intervention for alleviating symptoms of xylophagia. However, it is important to note that while patients may engage in pica for various reasons, these behaviors can prolong and exacerbate nutritional deficiency, specifically iron deficiency (15).

This case report highlights the manifestation of xylophagia in the context of iron deficiency anemia, adding to the growing body of evidence that links nutritional deficiencies to pica-related behaviors. Previous studies have documented similar associations, with several cases emphasizing the connection between iron deficiency and the compulsive ingestion of non-food items like paper. For instance, Shah et al. reported a 61-year-old woman with iron-deficiency anemia who presented with a colonic xylobezoar, underscoring the potential for serious gastrointestinal consequences of xylophagia in the setting of anemia (16). Similarly, Kurtz et al. described a 32-year-old woman with iron deficiency anemia who was found to have extensive toilet paper throughout her colon (17). Iron supplementation resulted in the resolution of these compulsions, indicating that iron deficiency plays a central role in triggering pica-like behaviors, including xylophagia. These cases underscore the importance of identifying and addressing underlying nutritional deficiencies as a critical component in the management—and potential reversal—of pica-related behaviors.

## Data Availability

The original contributions presented in the study are included in the article/supplementary material. Further inquiries can be directed to the corresponding author.
